# Habitat selection of Gould’s wild turkeys in southeastern Arizona

**DOI:** 10.1038/s41598-023-45684-1

**Published:** 2023-10-30

**Authors:** Erin E. Ulrey, Patrick H. Wightman, Nicholas W. Bakner, Byron R. Buckley, Nathan Fyffe, Brittany Oleson, Alex Smallwood, James R. Heffelfinger, Michael J. Chamberlain, Bret A. Collier

**Affiliations:** 1 Warnell School of Forestry and Natural Resources, University of Georgia, Athens, GA 30602 USA; 2 School of Renewable Natural Resources, Louisiana State University Agricultural Center, Baton Rouge, LA 70803 USA; 3 Arizona Game and Fish Department, 5000 Carefree Highway, Phoenix, AZ 85086 USA

**Keywords:** Ecology, Ecology

## Abstract

In semi-arid environments, resources necessary for survival may be unevenly distributed across the landscape. Gould’s wild turkeys (*Meleagris gallopavo mexicana*) are spatially restricted to mountainous semi-arid areas of southwestern United States and Mexico, and information on their distribution and habitat use is limited. We described how landcover type and topographical features influenced space use and habitat selection by Gould’s wild turkeys in southeastern Arizona. We used GPS data from 51 Gould’s wild turkeys to describe resource selection during 2016–2017 in southeastern Arizona, USA. We estimated home ranges and calculated resource selection functions using distance from landcover types, slope, aspect, and elevation at used locations and random locations within individual home ranges. Gould’s wild turkeys selected areas closer to pine forest and water. Likewise, Gould’s wild turkeys selected locations with moderate elevations of 1641 ± 235 m (range = 1223–2971 m), and on north and west facing slopes with a 10° ± 8.5 (range = 0.0–67.4°) incline. Our findings suggest that conserving portions of the landscape with appropriate topography and landcover types as described above will promote habitat availability for Gould’s wild turkeys.

## Introduction

Wildlife species preferentially select habitats that best meet their ecological needs^1–3^. In semi-arid environments, resources necessary for survival may be limited and unevenly distributed across the landscape. Vegetative communities in semi-arid environments are dynamic, fluctuating in quality due to variation in temperature, precipitation, and exploitation^4–7^. Thus, identifying critical habitats for various wildlife species across arid landscapes is important to prioritize management activities and direct conservation efforts.

The Gould’s wild turkey (*Meleagris gallopavo Mexicana*) originally occurred from central Mexico into southeastern Arizona and southwestern New Mexico^8,9^. Native Gould’s wild turkey were predominantly extirpated from most of the southwestern United States by the 1920s because of habitat degradation and unregulated hunting^8^. Starting in 1983 and continuing through 2018, the Arizona Game and Fish Department led translocation efforts to restore Gould’s wild turkeys throughout southeastern Arizona using birds from Mexico and populations from earlier translocation efforts^9–11^. Additional restoration efforts were conducted in 2014–2017, when Gould’s wild turkeys from Arizona were used to supplement populations in New Mexico (J. Heffelfinger, Arizona Game and Fish Department, personal communication).

Limited suitable habitat is thought to suppress Gould’s wild turkey population growth in the mountains of southeastern Arizona due to limited dispersal opportunities^12,13^. Previous research indicated that female Gould’s wild turkeys preferred pinyon pines (*Pinus spp.*), pinyon-ricegrass (*Achnatherum hymenoids*) and riparian habitats^14^. Riparian habitats may provide important roosting and foraging habitats^14–18^, but riparian habitats can be limited in mountainous, semi-arid areas that often experience drought and wildfire^14,19^.

Given the climatic extremes of the southwestern United States, topographical features can influence vegetative growth and suitability for various wildlife species^20,21^. Elevation gradients create climatic variation over relatively short distances^22,23^, whereas slope and aspect influence vegetative structure and species composition^24–29^. Within the range of Gould’s wild turkeys, moderate elevations (1,364–2,982 m) and north-facing slopes (10–65°) often support important oak (*Quercus* spp.), pine (*Pinus* spp.), juniper (*Juniperus* spp.), and manzanita (*Arctostaphylos pungens*) that provide roosting and foraging areas^14,15,30–32^. Conversely, xeric vegetation, at certain elevations, can dominate south-facing slopes which are hot and dry, and may be avoided by Gould’s wild turkeys^18,24,33^.

Our objectives were to describe how landcover type and topographical features influenced space use and habitat selection by Gould’s wild turkeys in southeastern Arizona. We hypothesized that Gould’s wild turkeys would select to be closer to specific landcover types, and that topographical features would influence selection. Specifically, we predicted that Gould’s wild turkeys would select areas closer to pine forest and water at moderate elevations on north facing slopes, as these areas presumably provide increased roosting^18^ and foraging^14,32^ opportunities compared to higher elevations and south facing slopes.

### Study area

**Figure 1:**
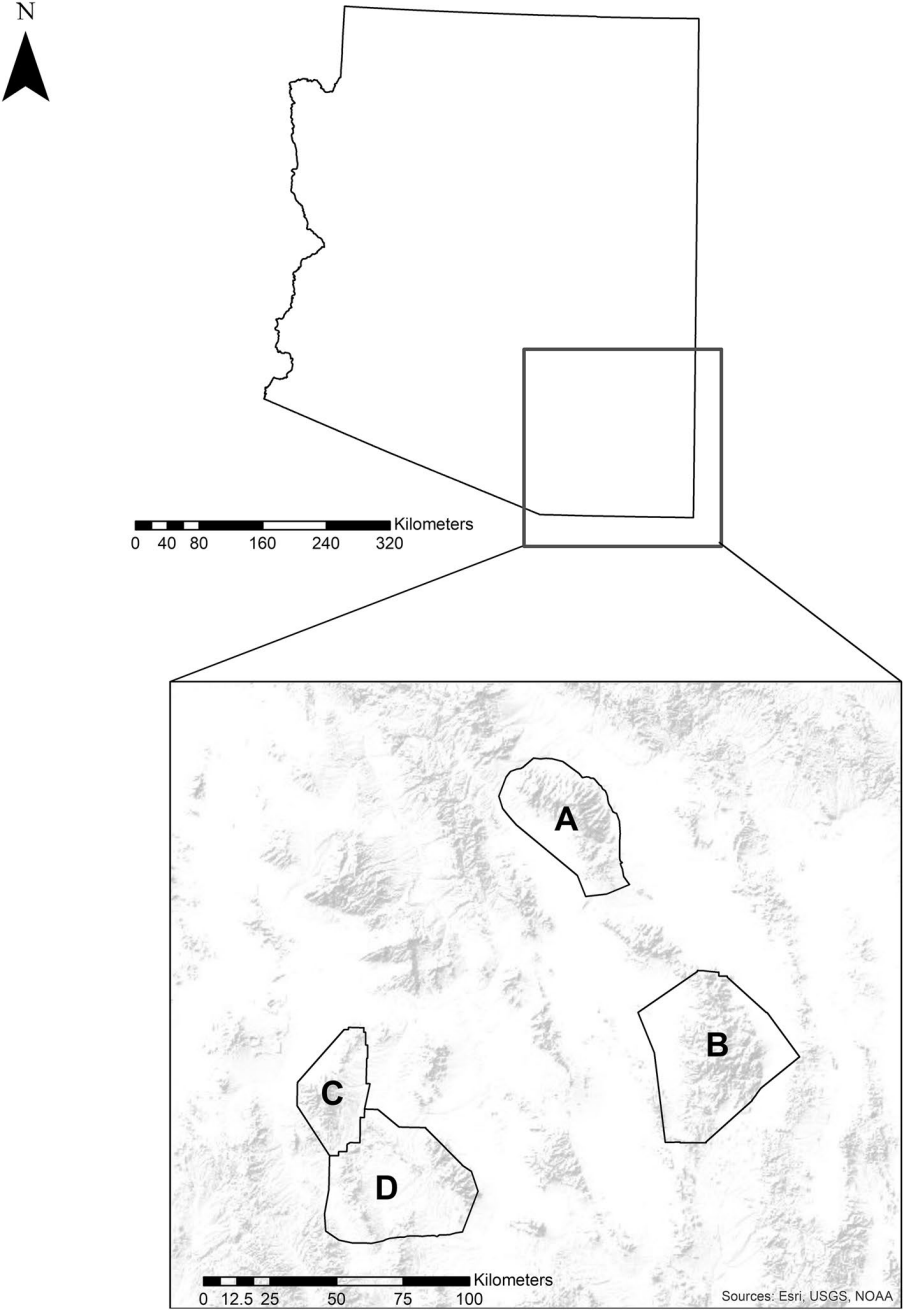
Map of study region in southeastern Arizona used to study Gould’s wild turkeys during 2016–2017, which included the (**A**) Pinaleño mountains, (**B**) Chiricahua mountains, (**C**) Santa Rita mountains, (**D**) Patagonia and Huachuca mountains.

We conducted our research in the Coronado National Forest in southeastern Arizona, which encompasses the sky islands extension of the Sierra Madre Occidental. Our specific study areas included the Pinaleño, Chiricahua, Huachuca, and Patagonia mountains located Graham, Cochise, and Santa Cruz counties (Fig. 1). All study sites consisted of semidesert grasslands of low to moderate shrub cover comprised of catclaw acacia (*Acacia greggii*), grama (*Bouteloua* spp.), needlegrass (*Achnatherum* spp.), Parry’s agave (*Agave parryi*), soaptree yucca (*Yucca elata*), and wheatgrass (*Elymus* spp.) found at elevations between 1,100 to 1,700 m. Madrean evergreen woodland including alligator juniper (*J. deppeana*), Arizona white oak (*Q. arizonica*), and Emory oak (*Q. emoryi*) were found at 1,200–2,300 m elevation. Petran montane conifer forest containing Douglas fir (*Pseudotsuga menziesii*), New Mexico locust (*Robinia neomexicana*), and Ponderosa pine (*P. ponderosa*) occurred at 2,000–3,050 m elevation, whereas Petran subalpine conifer consisting of Douglas fir and Engelmann spruce (*Picea engelmannii*) occurred at 2,450–3,800 m in elevation^34^. The highest of Arizona’s sky island mountain ranges was the Pinaleño Mountains, with a peak elevation of 3,267 m and base elevation of 974 m, compared to the Chiricauhas with a peak elevation of 2,974 m with a base elevation of 1,345 m. Similarly, the Huachuca Mountains had a peak elevation of 2,885 m with a base elevation of 1,524 m. The lowest sky island mountain range was Patagonia Mountains with a peak elevation of 2,200 m and a base elevation of 1,219 m^18^. Riparian corridors occurred along steep slopes and ravines, comprised of Arizona sycamore (*Platanus wrightii*) and Fremont cottonwood (*Populus fremontii*). Average annual temperatures ranged from 7.2° C to 16.7° C, respectively^35^ and average precipitation was approximately 579 mm^18^.

## Methods

### Capture and handling

We captured Gould’s wild turkeys with walk-in traps baited with cracked corn and peanuts during winter (January-March) 2016 and 2017^11,34,36^. Likewise, between 9–15 May 2017, Gould’s wild turkeys were captured on private property near Patagonia, Arizona in conjunction with an in-state translocation project^35^. We aged Gould’s wild turkeys based on presence of barring on the 9th and 10th primaries and sex of individuals was established based on the coloration of the breast feathers^37^. All individuals were equipped with a backpack-style global positioning system-very high frequency (GPS-VHF) transmitter^38^ (Biotrack, Wareham, Dorset, United Kingdom). We programmed transmitters to record hourly locations between 0500 and 2000, and 1 roosting location (23:58:58) until the battery died or the unit was recovered^39^. We immediately released non-translocated Gould’s wild turkeys at the capture location following processing. Translocated Gould’s wild turkeys were moved approximately 64 km to an area of similar topography and vegetation within our study area. Cohen^35^ indicated that after release, Gould's wild turkeys selected pine-juniper woodlands and riparian corridors immediately, and that there were no changes in roosting behavior. We remotely downloaded GPS data either via a fixed-wing aircraft with an ultra high frequency (UHF) handheld receiver (Biotrack) or ground based radio-tracking. All methods including turkey capture, handling, and marking procedures were performed in accordance with the relevant guidelines and regulations approved by the Louisiana State University Agricultural Animal Care and Use Committee.

### Environmental covariates

We examined selection of landscape features in relation to a set of environmental covariates relevant to the ecology of Gould’s wild turkey. We quantified topographical features (slope, aspect, elevation) using digital elevation models from the United States Geological Survey National Elevation Dataset (http://lta.cr.us.gs.gov/NED, accessed 10 Oct 2021)^34^. We converted aspect from a continuous variable to a categorical variable by binning degrees < 45 and ≥ 315 as north, ≥ 45 and < 135 as east, ≥ 135 and < 225 as south, and ≥ 225 and < 315 as west. We obtained year-specific, 30 m resolution spatial data on landcover from the Cropland Data Layer (Cropscape) provided by the National Agricultural Statistics Service (National Agricultural Statistics Service 2015). We recoded and combined landcover in R (v. 4.1.0; R Core Team, 2022) using package CropScapeR^40^ to create 6 unique landcover types (water, pine forest, hardwood forest, mixed pine-hardwood forest, barren, and shrub). We calculated the nearest distance from each turkey use and available points to each landcover type, using the Euclidean distance tool in ArcMap 10.8 (Esri, Redlands, CA, USA). We used landcover distance metrics for subsequent analysis instead of a classification or categorical approach^41^.

### Resource selection

We calculated 99% home ranges by fitting dynamic Brownian bridge movement models (dBBMMs) to the time-specific location data^39^ using package move^42^ in Program R (R Core Team 2022). We used an error estimate of 20 m, a moving window size of 7 locations, and a margin setting of 3 locations^39,43^.

We used resource selection functions (RSFs) to examine relationships between 3 topographical features (elevation, slope, aspect) and distance metrics from 6 landcover types (barren, hardwood forest, mixed pine hardwood forest, pine forest, shrub, water) to Gould’s wild turkeys use within individual home ranges (3rd-order selection) following a design III approach suggested by Manly^44^. We compared used points within individual home ranges to available points systematically sampled (every 3rd pixel, i.e., 90 m) within each home range^45^. We tested for collinearity between each of our covariates and excluded covariates using Pearson’s correlation with a *r* > 0.60^46^. We fit a single global (i.e., including all covariates) generalized linear mixed model (GLMM) to include a random intercept for each individual turkey, with a binomial response distribution (logistic regression) and logit link to the used-available data for Gould’s wild turkeys^44,47^. We included a random intercept for each individual turkey to account for individual variation in response to available conditions and unbalanced sampling^48^. We used the lme4 package in program R^49^ with a binary (0 = available, 1 = used) response variable to model resource selection. We rescaled all variables by subtracting their mean and dividing by their standard deviation prior to modeling^50^. We did not use model selection techniques to rank candidate models because the relative effect of all covariates were of interest. To assess how well our RSF model explained the data, we used area under the receiver-operating characteristic curves (AUC) calculated with the pROC package in program R^51^. An AUC value of 0.5 indicated the model provided estimates no better than random predictions, but values greater than 0.7 indicated a better model fit with more accurate predictions.

### Ethical approval

Turkey capture, handling, and marking procedures were approved by the Louisiana State University Agricultural Animal Care and Use Committee (Protocol number A2015-07).

## Results

We captured 45 Gould’s wild turkeys (2016: 19 females, 1 male; 2017: 25 females) during January-March 2016 and 2017, and captured and translocated 17 individuals (6 females, 11 males) in May 2017. We censored 11 individuals (2016: 6 females; 2017: 1 translocated females, 3 translocated males, and 1 non-translocated female) due to transmitter failure or post-release mortality. We created 51 individual Gould’s wild turkeys home ranges (2016: 13 females and 1 male; 2017: 29 females and 8 males) from 156,184 GPS locations. Transmitters collected data for an average of 7 months (SD ± 2, range 1–15), but because of varying deployment dates we had GPS locations spanning all 12 months (Table S1). The average 99% home range size was 2,001 ha (SE ± 239; range 143–7,518).

**Figure 2:**
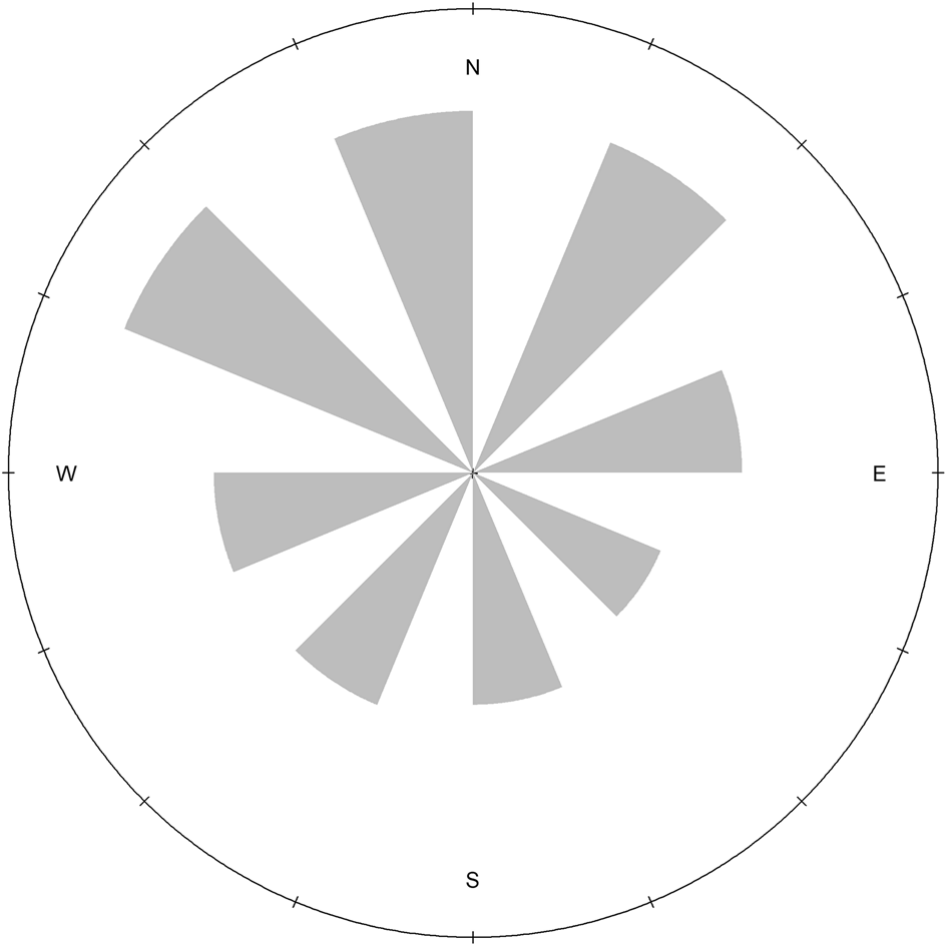
Circular plot of aspect (°) for Gould’s wild turkey (*Meleagris gallopavo mexicana*) GPS locations in Coronado National Forest in southeastern Arizona, USA, 2016–2017. Gray bars represent the distribution of data.

**Figure 3:**
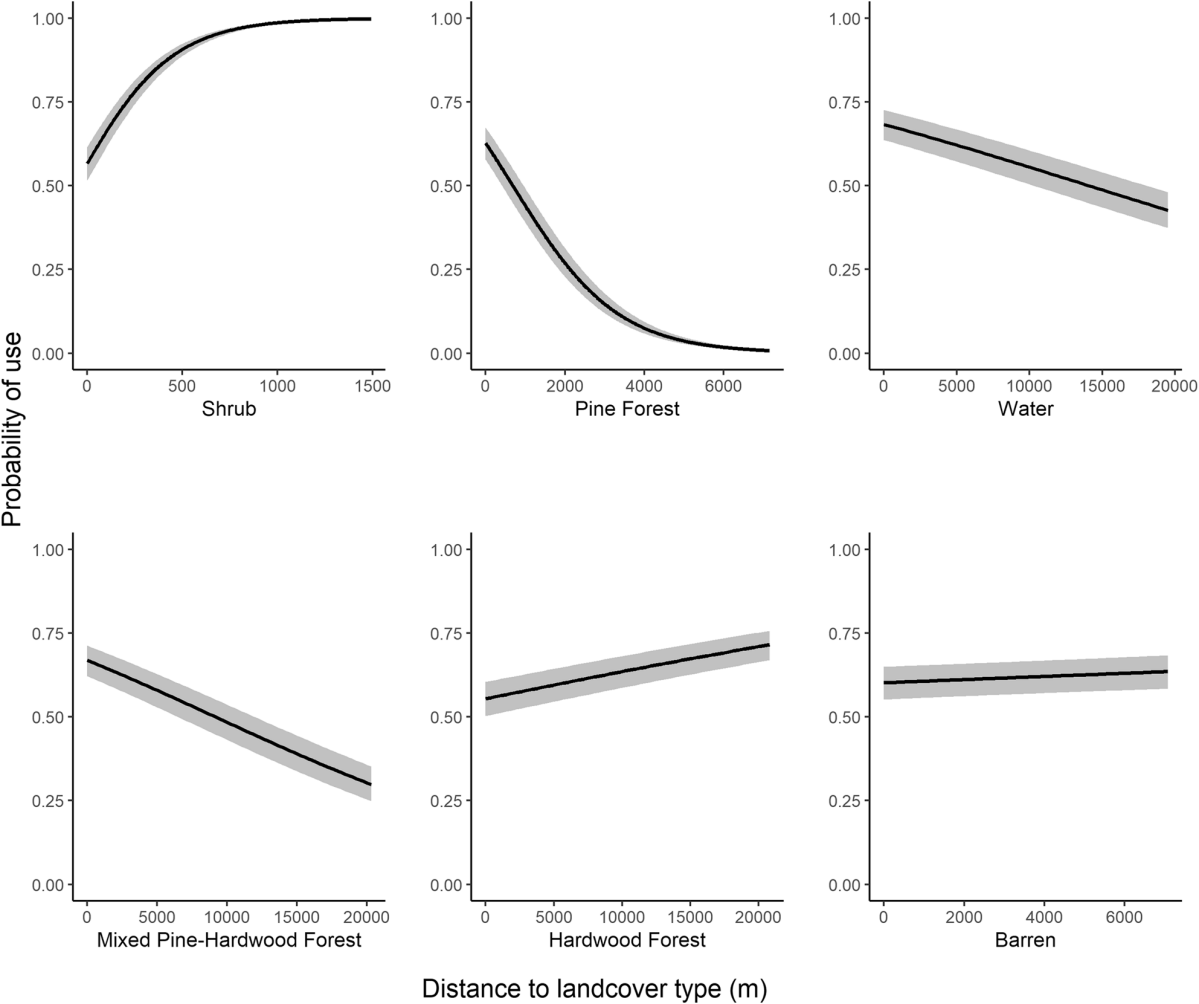
Predicted probability of habitat selection for landcover type (solid line) with 95% confidence intervals (gray ribbon) from the global model for Gould’s wild turkeys (*Meleagris gallopavo mexicana*) in Coronado National Forest, southeastern Arizona, USA, 2016–2017.

**Figure 4:**
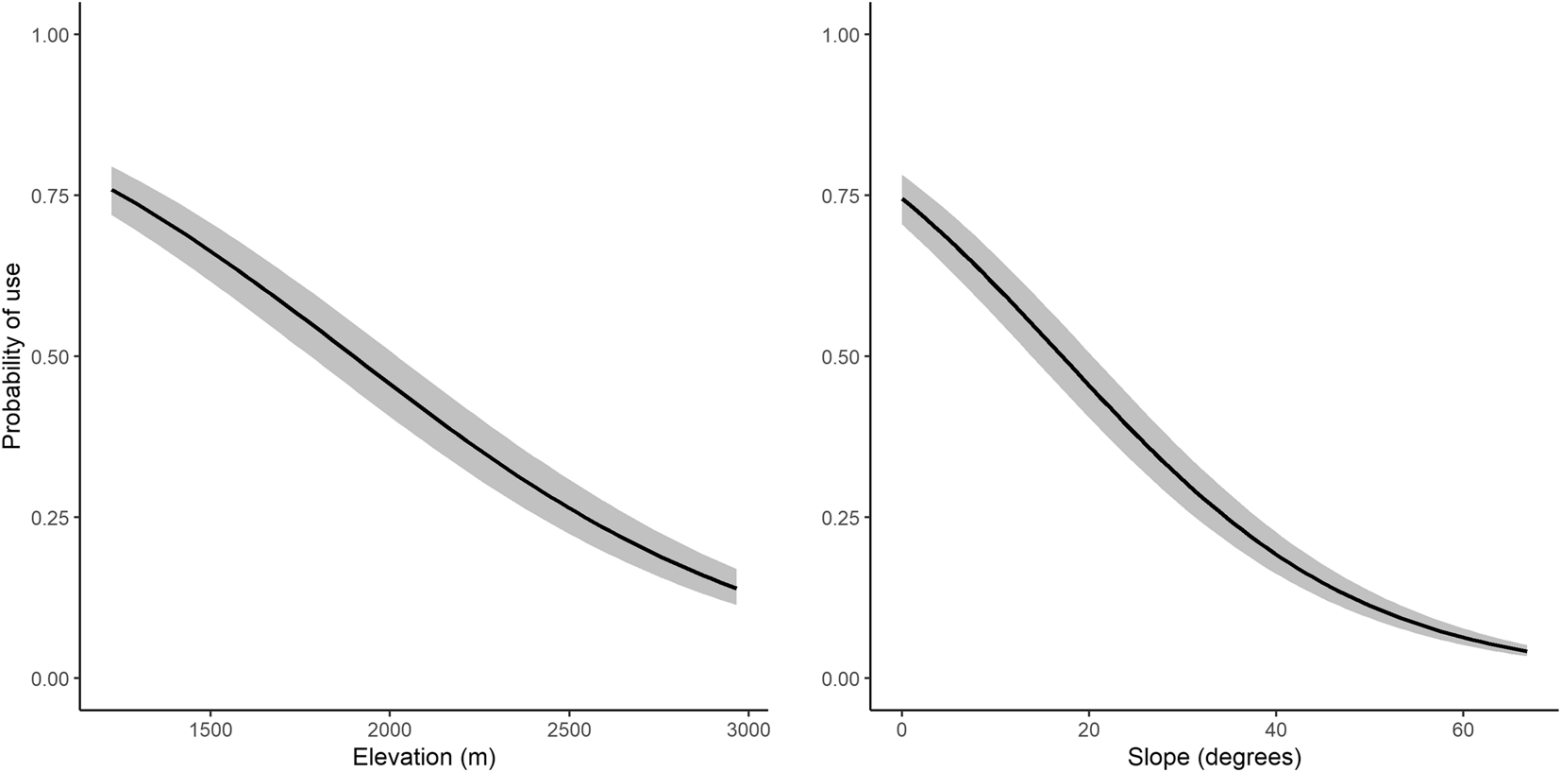
Predicted probability of habitat selection relative to elevation and slope (solid line) with 95% confidence intervals (gray ribbon) from the global model for Gould’s wild turkeys (*Meleagris gallopavo mexicana*) in Coronado National Forest, southeastern Arizona, USA, 2016–2017.

In our global model, parameter estimates for landscape characteristics and landcover types were statistically different from zero except for east aspect (Table 1). We kept all variables in the global model as none were correlated with other variables. Selection probability was positively associated with north and west facing aspects, and Gould’s wild turkeys avoided south facing aspects (Table 1, Fig. 2). Gould’s wild turkeys avoided hardwood forest, shrub, and barren habitat (Table 1, Fig. 3), but selected for water, pine forest, and mixed hardwood pine forest (Fig. 3). As elevation and slope increased, probability of selection decreased (Fig. 3). Gould’s wild turkeys selected for an average elevation of 1,641 ± 235 m (range = 1,223–2,971 m; Fig. 4), with a mean slope of 10 ± 8.5° (range = 0.0–67.4°; Fig. 4). The AUC estimate was 0.74, indicating suitable accuracy of our model.

**Table 1:** Parameter estimates predicting habitat selection of Gould’s wild turkeys (*Meleagris gallopavo mexicana*) in Coronado National Forest, southeastern Arizona, USA, 2016 and 2017.

Parameters	β estimate	SE	*Z*	*P*-value
Aspect East	0.05	0.104	0.47	0.64
Aspect North	0.39	0.011	34.08	< 0.01
Aspect South	− 0.57	0.012	− 45.41	< 0.01
Aspect West	0.05	0.012	4.09	< 0.01
Elevation	− 0.40	0.010	− 40.03	< 0.01
Hardwood	0.13	0.009	13.14	< 0.01
Pine	− 0.20	0.005	− 38.75	< 0.01
Slope	− 0.53	0.005	− 100.02	< 0.01
Barren	0.02	0.006	3.22	< 0.01
Shrub	0.32	0.006	50.30	< 0.01
Water	− 0.19	0.010	− 18.16	< 0.01
Mixed pine-hardwood forest	− 0.20	0.011	− 18.29	< 0.01

## Discussion

Our findings support the hypothesis that landcover type and topographical features influence resource selection of Gould’s wild turkeys. We noted a positive relationship between Gould’s wild turkeys use and north facing slopes, but a negative relationship with south facing slopes, which was consistent with the roost site selection analysis by^18^. In addition, we found evidence consistent with previous studies documenting the importance of pine forests and avoidance of shrub habitat and bare ground^32^. Conversely, our findings suggest that Gould’s wild turkeys preferred lower elevations than those reported in previous studies^11,14,32^. The average home range size for our individuals was smaller than home range sizes reported in other studies, although our home range estimates may be biased slightly high as some of the translocated birds may still be conducting searching activities^35^. We offer that the resultant differences we found were likely due to the use of finer scale spatial data obtained from GPS transmitters compared to earlier studies that relied on VHF transmitters and triangulation^38^.

Resource availability across a landscape can influence how species select habitats^20,52^, and Gould’s wild turkeys occur in arid environments where resources are often less abundant^53,54^. Our results indicated that Gould’s wild turkeys selected for locations at moderate elevations (1,223–2,971 m). Elevations of 1,000–2,300 m were associated with oak and pine-oak forest, juniper, and riparian corridors across our study areas. Juniper and mature pine-oak forests with open canopies provide open-herbaceous understories, which create food sources for Gould’s wild turkeys^14^. Likewise, large mature pines provide turkeys with suitable roost sites^18^, demonstrating the importance of pine forests near suitable areas for foraging.

In the southwestern United States, north-facing slopes are cooler relative to south-facing slopes because they receive less sunlight and offer more shade and moisture, which influences the distribution and growth patterns of vegetation^55,56^. Hence, north-facing slopes support more productive vegetative communities, that are important to Gould’s wild turkeys^14^. Conversely, south-facing slopes are drier and warmer^24,33^, and are typically dominated by barren and shrub landcover types that turkeys use less^15,32,57^. Furthermore, Gould’s wild turkeys may seek shade and cooler areas associated with north facing slopes to maintain appropriate body temperatures^58,59^. We suggest future research examine how thermal conditions may influence resource selection by Gould’s wild turkeys^60,61^.

Our findings provide additional support for the conservation and restoration of forest communities on north and west facing slopes across the southern sky islands of southern Arizona, in particular mixed hardwood pine forests that provide important resources for Gould’s wild turkey. Likewise, our findings provide substantive evidence that pine and mixed hardwood pine forests at lower to moderate elevations (1223–2971 m) are important to sustainability of Gould’s wild turkeys. Management activities should prioritize retention of pine forests near riparian corridors because they provide important roosting and foraging habitat in semi-arid landscapes.

### Supplementary Information

Supplementary Table S1.

## Data Availability

The datasets generated during and/or analyzed during the current study are available from the corresponding author on reasonable request.
