# Supplementary material for: Habitat selection of Gould’s wild turkeys in southeastern Arizona
Source: Sci Rep. 2023 Oct 30;13:18639. doi: 10.1038/s41598-023-45684-1 (PMC10616159; doi:10.1038/s41598-023-45684-1)
Supplement: Supplementary file 1 — Supplementary Table S1. [file 41598_2023_45684_MOESM1_ESM.pdf]

Table S1. Number (count) of Gould’s wild turkeys (*Meleagris gallopavo mexicana*) with working transmitters for each month of the year by sex and if the individual was translocated.

Translocated	Sex	Month	Count
No	F	January	20
No	F	February	32
No	F	March	37
No	F	April	37
No	F	May	37
No	F	June	35
No	F	July	33
No	F	August	25
No	F	September	10
No	F	October	6
No	F	November	6
No	F	December	6
No	M	January	1
No	M	February	1
No	M	March	1
No	M	April	1
No	M	May	1
Yes	F	January	1
Yes	F	February	1
Yes	F	March	1
Yes	F	April	1
Yes	F	May	6
Yes	F	June	5
Yes	F	July	5
Yes	F	August	4
Yes	M	May	8
Yes	M	June	8
Yes	M	July	7
Yes	M	August	7
Yes	M	September	7
Yes	M	October	7
Yes	M	November	7
Yes	M	December	6
